# A review of weight loss and sarcopenia in patients with head and neck cancer treated with chemoradiation

**DOI:** 10.1186/s41199-016-0010-0

**Published:** 2016-08-17

**Authors:** Shrujal S. Baxi, Emily Schwitzer, Lee W. Jones

**Affiliations:** 1 Head and Neck Oncology Service, Department of Medicine, Memorial Sloan Kettering Cancer Center, 300 East 66th Street, Rm 1459, New York, NY 10065 USA; 2 Department of Medicine, Memorial Sloan Kettering Cancer Center, 1275 York Ave, New York, NY 10065 USA

**Keywords:** Head and neck cancer, Sarcopenia, Chemoradiation, Quality of life, Cardiopulmonary fitness, Nutrition

## Abstract

**Background:**

Concurrent chemotherapy and radiation (CTRT) improves disease-free survival in locally advanced head and neck cancer but is associated with numerous acute and chronic toxicities resulting in substantial alterations in body mass and composition. We aim to summarize the current evidence on body composition changes experienced by patients undergoing CTRT, examine the impact of these changes on clinical outcomes and address potential interventions aimed at mitigating the loss.

**Main Body:**

Loss of 20 % of pre-CTRT weight predicts poorer treatment tolerance and 30-day mortality. While clinical practice focuses on body weight, emerging data indicates that CTRT causes profound adverse changes in lean body mass (sarcopenia). Higher prevalence of sarcopenia predicts poorer disease-free survival as well as overall survival, lower quality of life and functional performance. The magnitude of CTRT-induced sarcopenia is the equivalent to that observed in a decade of aging in a healthy adult. Alterations in body composition are only explained, in part, by decreased caloric intake; other significant predictors include body mass index, stage, and dysphagia. Lifestyle interventions aimed at preventing loss of whole-body and especially lean mass include nutritional counseling, nutritional supplements, dietary supplements and exercise training. Personalized nutritional counseling has been associated with improvement in quality of life, while the benefits of feeding tube placement are inconsistent. There are inconsistently reported benefits of resistance training in this population.

**Conclusion:**

Patients with head and neck cancer undergoing CTRT therapy experience dramatic shifts in body composition, including sarcopenia, which can negatively impact clinical outcomes. Efforts to understand the magnitude, clinical importance and mechanisms of sarcopenia are needed to inform a more personalized approach to mitigating the body composition changes associated with CTRT.

## Background

Head and neck squamous cell carcinoma (HNSCC) is a group of diseases arising from the upper aerodigestive tract including the oral cavity, pharynx and larynx. There are an estimated 59,000 cases of HNSCC diagnosed annually in the US, representing 3 % of all newly identified cancers [1, 2]. Despite remaining relatively consistent in overall incidence, the epidemiology of HNSCC has changed over the last three decades, most notably in Western countries [3]. Once considered a disease resulting mostly from chronic exposure to tobacco and alcohol, it is now recognized that there are other causative factors for HNSCC. One of the fastest growing subsets of patients are those diagnosed with HNSCC tumors arising from the oropharynx secondary to human papilloma-virus (HPV) [4]. Patients with HPV-positive tumors have a striking improvement in survival compared with patients with historical, HPV-negative tumors with 3-year survival of 82 % versus 57 %, respectively (*p* < 0.001) [5]. Patients with HPV-associated tumors also appear to have a lower risk of developing second primary malignancies, resulting in longer long-term survival [5, 6]. Changing epidemiology along with treatment advances have resulted in a rise in the number of survivors of HNSCC who are expected to live well beyond their cancer diagnosis and treatment. Efforts are underway to better understand the toxicities associated with treatment and implement strategies aimed at improving the long-term quality of life and survival in this emerging cohort of cancer survivors [7].

## Main text

Approximately 70 % of patients with HNSCC will present with locally advanced disease and many will be treated with concurrent chemotherapy and radiation (CTRT). Concurrent chemotherapy and radiation is associated with significant in-field and systemic toxicities including mucositis, dysphagia, odynophagia, nausea, vomiting, anorexia, fatigue and dysgeusia resulting in difficulty eating [8–10]. Furthermore, many patients present with symptomatic tumors that lead to difficulty eating prior to the initiation of treatment, with most patients experiencing a loss of more than 5 % of pre-treatment body weight in the 6 months around CTRT [11–14]. In part, this has been exacerbated by a change in resting energy expenditure, which furthers the loss of lean body mass seen during and immediately after treatment [11, 15]. Predictors of excessive weight loss during treatment include higher weight at baseline, dysphagia at diagnosis, and higher stage tumors [16]. More specifically, a recent study from Denmark reported that patients with a body mass index (BMI) ≥ 25 are three times more likely to lose more than 5 % of baseline weight than patients with BMI < 25 (*p* < 0.0001) [12].

Weight loss has been negatively associated with tumor control and survival outcomes in HNSCC [13]. For example, patients with early significant weight loss (defined as >20 % loss from baseline) are more likely to die from a treatment-related complication within 30 days of completing CTRT compared to those who do not (*p* = 0.029). In a secondary analysis from the phase III SAKK 10/94 trial of hyperfractionated radiation versus cisplatin and standard fraction radiation, Ghadjar and colleagues found that weight loss during treatment on either arm was common, but only weight loss experienced before treatment was associated with decreased time to treatment failure, disease free survival (DFS), and overall survival (OS) (*p* < 0.05 for all); weight loss occurring during treatment was not associated with survival outcomes [14]. The results from this study highlight two important aspects of weight loss and HNSCC. First, patients who are already losing weight at diagnosis are at a disadvantage and may be presenting with more aggressive disease at baseline. Second, while weight loss during treatment is common, it is not universally prognostic. One explanation for this discrepancy is that in some patients the weight loss is caused of decreased caloric intake, while in others weight loss is a manifestation of cachexia, or a syndrome of dysregulated catabolism and anabolism [17]. Historically, high-risk patients would have a percutaneous endoscopic gastrostomy (PEG) placed prior to starting treatment in order to prevent decreased caloric intake, but more recently there has been movement away from using feeding tubes due to concerns about delayed recovery of swallowing post-CTRT [18–20]. Interestingly, the placement of a PEG, or increased caloric intake alone, does not alleviate all the weight loss experienced during CTRT [12, 19, 21].

The weight loss experienced by patients with HNSCC undergoing CTRT is more specifically a change in body composition. Body weight or body mass is composed of fat, bone, water and lean body mass and the loss of lean body mass accounts for more than 70 % of weight lost during CTRT [15, 22]. Loss of lean body mass in cancer is mostly explained by sarcopenia, or loss of skeletal muscle [23]. Patients with HNSCC undergoing CTRT are often losing more than 5 % of their total muscle mass in less than 6 months time which is equivalent to the amount of muscle mass lost in the average, inactive adult over the course of a decade [24–26]. Skeletal muscle is the largest organ in the body and makes up approximately 50 % of total body weight and is essential for movement, strength, balance, body temperature regulation and respiration. Maintaining muscle mass involves a balance of protein breakdown (catabolism) and muscle synthesis (anabolism) and is tightly regulated through a network of external signaling pathways leading to intracellular gene transcription [22]. Lean body mass, mostly composed of muscle, can be measured using both validated direct and indirect methods. Examples of possible techniques include bioelectrical impedance (BIA), measurement of air displacement plethysmography, dual energy x-ray (DXA, and cross-sectional imaging on computed tomography (CT) or magnetic resonance imaging (MRI) [27].

Importantly, loss of lean body mass is associated with poorer treatment tolerance and worse cancer outcomes [28–30]. For example, in patients receiving systemic chemotherapy for colorectal cancer, loss of ≥ 9 % muscle mass during a 3 month period was independently predictive of lower survival at 6 months compared to patients who had < 9 % loss, 33 % versus 69 %, respectively, despite no difference in treatment modifications [31]. Similar associations have been described in patients with HNSCC. Grossberg and colleagues recently reported that in a cohort of 190 patients with HNSCC treated with definitive radiation, decreased overall survival was associated with baseline sarcopenia (Hazard Ratio (HR) 1.92, 95 % confidence interval [CI] 1.19–3.11) and post-radiation sarcopenia (HR 2.03, 95 % CI 1.02–4.24). However, in this analysis, weight loss alone, without associated loss of skeletal muscle, was not associated with worse clinical outcomes [32]. Further, when they evaluated by subsites, skeletal muscle depletion was associated with decreased survival in patients with non-oropharyngeal cancer (*n* = 51) but not in patients with oropharyngeal cancer (*n* = 139). This is a retrospective study and while causation cannot be gleaned, the results highlight that patients with more significant loss of skeletal muscle are experiencing poorer clinical outcomes. Further, it also highlights that patients with HNSCC are a heterogenous group and more research is necessary to understand what, and if any, relationship exists between change in body composition and oncologic outcomes. Emerging data suggests that patients with HPV-related oropharyngeal cancer, generally a healthier and younger population at diagnosis, are also experiencing significant alterations in body composition as a result of CTRT, but that this may not negatively impact the expected excellent clinical outcomes, at least not at early follow-up [33].

Sarcopenia is not only relevant for oncologic outcomes, but is predictive of worse survival in the general, non-cancer, and population as well. It is associated with numerous negative outcomes, including an impaired stress response, frailty, functional impairment, lower quality of life and decreased overall survival [34–37]. As such, acute sarcopenia, could then explain, at least in part, the dramatic drop in quality of life experienced by even the most functional patients at baseline who undergo CTRT for HNSCC [38]. In a large cross-sectional study in people over the age of 65, sarcopenia predicted for three times more functional impairment in females and two times more functional impairment in males compared to age- and sex-matched participants with normal lean body mass [39]. Further, a large portion of age-associated decline in cardiopulmonary fitness, measured by VO_2peak_, is also explained by sarcopenia [40]. Low cardiorespiratory fitness is an important precursor for premature mortality regardless of underlying cardiovascular risk factors [41]. Altogether, this highlights the need for studies to better understand the impact of sarcopenia sustained during CTRT and its impact on cardiorespiratory fitness and subsequent non-oncologic mortality in patients with HNSCC.

The current strategies employed to combat metabolic derangements experienced by patients during CTRT generally focus on weight maintenance. Patients are encouraged to eat “as many calories as they can” and when food gets harder to swallow, supplementing it with either oral or parenteral nutrition via a PEG [42]. However, decreased caloric intake is probably only partially responsible for sarcopenia and simply increasing calories may not address loss of muscle mass. In a review of sarcopenia and cachexia in patients with all stages of HNSCC, Couch and colleagues describe the dysregulated metabolism and the resulting sarcopenia seen in patients with HNSCC, suggesting that nutrition alone is not enough to counteract the impact of body composition [21]. In spite of this, numerous studies have tried to optimize nutritional support in patients undergoing CTRT. Clinical trials have evaluated the benefit of individualized nutrition counseling, oral supplementation, and the right type and route of supplemental nutrition. In a systematic review on the effect of nutritional interventions on weight, quality of life and mortality in HNSCC patients treated with RT or CTRT, individualized dietary counseling was beneficial on quality of life, but the impact of tube feedings was not conclusive [43]. For example, in a prospective study of patients with HNSCC undergoing RT, Ravasco and colleagues found that nutritional counseling with regular foods was superior to only adding nutritional supplements in maintaining quality of life during and at 3 months post-RT, but the impact of these interventions on weight was not reported [44]. In a large retrospective review of patients on RTOG 90-03, investigators performed a secondary analysis of patients with HNSCC treated with four different radiation strategies, Rabinovitch and colleagues found that beginning nutritional supplementation before starting RT was a negative prognostic indicator for locoregional failure and death, HR 1.47, 95 % CI 1.21–1.79, *p* < .0001 and HR 1.41, 95 % CI 1.19–1.67, *p* < .0001, respectively [45]. Given the retrospective nature of this analysis, nutritional supplement was likely initiated pre-RT in patients who presented with weight loss at diagnosis. This suggests that patients with baseline weight loss have a worse clinical outcomes and not that nutritional supplement itself is having a negative impact on outcomes.

Integration of optimal nutritional support into clinical practice for HNSCC patients has been impeded due to the inconsistent methodology across studies, measuring various time points and outcomes. In response, a group of investigators published a review of the current state of the science on nutrition and malnutrition in HNSCC and provided a set of consensus criteria for implementation and definitions in studies on nutrition in this population [46].

There is growing interest in using pharmacologic supplements to combat cancer-related sarcopenia and cachexia, including amino acids, anabolic steroids, anti-inflammatory agents, and ghrelin-analogues, to counteract the deranged metabolism experienced by cancer patients. Most research in this space has been completed in patients with metastatic disease, often non-small cell lung cancer [44]. One agent of interest in HNSCC patients is eicosapentaenoic acid (EPA), which is an alpha-3-omega fatty acid found in fish oil. In vitro, EPA has been shown to mitigate the lipolysis and inflammatory underpinnings of cachexia [47, 48]. Patients with HNSCC undergoing primary surgical approach did show short-term benefit in loss of muscle mass with EPA, but longer-term follow is needed [49]. A Cochrane review on the use of EPA in cancer cachexia was completed in 2007 and recommended against regular use of EPA [50]. An alternative approach recently explored is anamorelin, an oral ghrelin mimetic. Ghrelin is a ligand for the growth hormone secretagogue receptor and leads to release of growth hormone. There have been two large phase III, double blind placebo-controlled trials of anamorelin in patients with cachexia resulting from unresectable lung cancer. The two studies included a combination of more 900 patients and reported improvement in body weight and anorexia with anamorelin but no improvement in overall survival or strength [51]. Hopefully, pharmacologic interventions will follow a better understanding of the exact mechanism of sarcopenia in patients with HNSCC undergoing CTRT.

Another intervention with the potential to counteract the negative effects of CTRT on body composition is exercise. Exercise has emerged as a promising component of cancer care and has been shown to improve clinical and survival outcomes [52–54]. In a large systematic review and meta-analysis, Speck and colleagues reported favorable changes of exercise on body composition, cardiopulmonary fitness, muscular fitness, psychological well-being, flexibility and quality of life for cancer patients both during and after treatment. While this study included 82 trials, few of the included studies focused on patients with HNSCC [55]. Of those that did, the majority used a progressive resistance training program to improve muscle quantity, quality and function. As sarcopenia and impaired physical functioning remain primary concerns for these patients, this becomes clinically important. Indeed, randomized studies from both breast and prostate cancer patients have shown that resistance training can increase lean muscle mass compared to the control (i.e., sedentary) group [52, 54, 56, 57]. This contrasts to aerobic (or endurance) exercise training, which primarily focuses on improving one’s peak oxygen consumption (VO_2peak_) – another independent predictor of survival in both oncologic and non-oncologic populations alike. Together, aerobic and resistance exercise training, are complementary to both the cardiovascular and musculoskeletal system.

Against this background, exercise is increasingly being explored as a potential method to combat the negative impact of HNSCC and its treatment on patients. A prior cross-sectional study demonstrated that HNSCC patients get less than the recommended level of activity at all time points; specifically, only 30.5 % are active at baseline, falling to only 8.5 % of patients at the end of treatment [58]. This demonstrates the great potential for exercise to improve outcomes in this currently inactive group. There is good data available on the benefit of therapeutic exercises for shoulder dysfunction or trismus resulting from fibrosis following treatment, but the systemic impacts of exercise in HNSCC are less well understood. We performed a review and summarized the data from studies on progressive resistance training in patients treated for locally advanced HNSCC (Table 1). There was considerable heterogeneity in the study methodology, differing in the type and timing of exercise intervention, as well as the outcomes measured. Regardless, it is clear from the control arms, when available, that there is a negative impact of no intervention [59, 60]. Much like the limitations of current literature in nutritional interventions for HNSCC, there is a need to standardize research in exercise interventions including the type, timing, frequency and duration of studied regimens. Only then can the results from one study be compared to the next and provide measurable, reproducible and generalizable interventions that are relevant to clinical practice.

**Table 1:** Prospective Studies on resistance training in patients with head and neck cancer

Author	Subjects	Exercise Intervention	Major Findings
McNeely et al. 2008	52 HNSCC patients after neck dissection	12 week supervised PRT (2-3x/week) versus standard of care.	Adherence: 95 % for PRT group and 87 % for control group.
			Outcomes: PRT was superior to standard of care for improving shoulder pain and disability (*p* < 0.001), upper extremity strength (*p* < 0.001), and upper extremity endurance (*p* < 0.039)
Lonbro, DAHANCA 25A 2013	30 HNSCC patients after curative radiotherapy +/- chemotherapy	12 week partially supervised PRT (2-3x/week) with or without a seven day creatinine load	Adherence: 97 % in those that completed the study with a completion rate of 70 %
			Outcomes: Addition of creatinine to PRT did not improve lean body mass (*p* = 0.07). Regardless of nutritional intervention, improvement noted after PRT in lean body mass and maximal isometric and isokinetic muscle strength.
Lonbro, DAHANCA 25B 2013	41 HNSCC patients after curative radiotherapy +/- chemotherapy	24 week study of early versus delayed 12 week supervised PRT (2-3x/week).	Adherence: Not reported
			Outcomes: Increase in lean body mass by 4.3 % and 4.2 % in early versus delayed PRT. Improvement larger than change after self-chosen physical activity (*p* < 0.005). Regardless of PRT start-up time, the odds ratio of increasing LBM by more than 4 % after PRT was 6.26 (*p* < 0.05).
Rogers et al. 2013	15 HNSCC patients during radiation therapy	12 week supervised PRT (2x/week) for 6 weeks then at home PRT (2x/week) versus standard of care.	Adherence: 83 % for supervised exercise and 62 % for exercise telephone counseling.
			Outcomes: PRT improved in fatigue and quality of life at 6 weeks versus control. Chair rise time (seconds) improved at 6 and 12 weeks in PRT arm versus standard of care (-1.6 vs 0.4 respectively, *p* < 0.05).
Samuel et al. 2013	48 HNSCC patients during CRT	6 week supervised general exercise program (5-6x/week) versus routine physical activity encouragement.	Adherence: not recorded
			Outcomes: Increased 6MWD in theintervention arm, decreased in the control arm with a 138 m difference between groups (*p* < 0.001).
Capozzi et al. 2016	60 newly diagnosed HNSCC patients during RT or CRT	24 week study of immediate versus delayed with a 12 week supervised PRT (2x/week) and nutrition intervention.	Adherence: 45.2 % in immediate group and 61.5 % in delayed group.
			Outcomes: No difference in lean body mass or percentage body fat at 24 weeks.

*AE* adverse event, *CRT* concurrent chemoradiotherapy, *HNSCC* head and neck squamous cell cancer, *PRT* progressive resistance training, *RT* radiation therapy, *6MWD* six minute walk distance

## Conclusions

HNSCC survivors remain at a substantial risk for long-term disability and early mortality despite achieving initial tumor control [58, 61]. Younger patients are now presenting with HNSCC due to the epidemic of HPV-related oropharyngeal cancer and have an expected better overall prognosis. As these patients live longer past diagnosis, maintaining quality of life and long-term survival is paramount. Longitudinal studies are required to measure the rates of recovery of weight and lean body mass and then in turn, discover new strategies to assist patients as they try to return to baseline functional capacity after aggressive CTRT therapy. Patients with HNSCC are unique in the challenges they face, and the applicability of studies performed in other cancer populations may not be directly relevant to these patients; standardized nutritional and exercise interventions are needed for HNSCC.

## Abbreviations

CI, confidence interval; CTRT, concurrent chemotherapy and radiation; DFS, disease free survival; HNSCC, head and neck squamous cell carcinoma; HPV, human papillomavirus; HR, hazards ratio; OS, Overall survival; PEG, percutaneous endoscopic gastrostomy; RT, radiation
